# Conditioned pain modulation in drug-naïve patients with de novo Parkinson’s disease

**DOI:** 10.1186/s42466-019-0029-x

**Published:** 2019-08-26

**Authors:** Wiebke Grashorn, Odette Fründt, Carsten Buhmann, Nathalie Wrobel, Katharina Schmidt, Ulrike Bingel

**Affiliations:** 1 Department of Neurology, University Medical Center Hamburg – Eppendorf, Martinistr. 52, 20246 Hamburg, Germany; 2 Department of Neurology, University Hospital Essen, University Duisburg-Essen, Hufelandstrasse 55, 45147 Essen, Germany; 3 Karolinska Institutet, 171 77 Stockholm, Sweden; 4 Erwin L. Hahn Institute for magnetic resonance imaging, Essen, Germany

**Keywords:** CPM, Descending inhibition, Neurodegeneration, Dopamine

## Abstract

**Background:**

Pain is highly prevalent in patients with Parkinson’s disease (PD), but underlying pathophysiological mechanisms are largely unclear. In many chronic pain syndromes deficits in endogenous pain inhibition have been detected that can be assessed using conditioned pain modulation paradigms. Previous studies employing this approach in medicated PD patients did not find abnormal pain inhibition. However, these results might have been confounded by residual dopaminergic medication.

**Methods:**

An established conditioned pain modulation paradigm was used in 17 drug-naïve de novo PD patients and 17 healthy age and gender-matched controls. We tested i) whether conditioned pain modulation responses differed between the patient and control group and ii) whether pain inhibition differed between PD subtypes.

**Results:**

PD patients and healthy controls did not differ in their conditioned pain modulation responses. Furthermore, there were no significant differences in CPM responses depending on the PD subtype. However, at a descriptive level, tremor-dominant patients showed a tendency for better descending pain inhibition compared to akinetic-rigid and mixed type patients.

**Conclusions:**

In this first study investigating conditioned pain modulation in de novo PD patients, we found no additional impairment in descending pain modulation besides the known age-related decline. Our findings indicate that mechanisms other than an impaired descending inhibition contribute to high pain prevalence rates in PD and suggest that mechanisms underlying pain may differ between PD subtypes.

**Electronic supplementary material:**

The online version of this article (10.1186/s42466-019-0029-x) contains supplementary material, which is available to authorized users.

## Introduction

Pain is a highly prevalent symptom in patients with Parkinson’s disease (PD) and affects up to 90% of the patients [3, 8]. It significantly impairs patients’ quality of life [42, 44] and has been reported to precede motor symptoms [50]. Charcot already described pain in PD in 1878 [18], yet only little is known about its underlying pathophysiological mechanisms.

The susceptibility to acute and chronic pain is supposed to depend on the balance of activity in ascending and descending pain pathways [33, 38, 51]. The descending pain control system can modulate pain by either inhibiting or facilitating nociceptive processing [33, 38]. Because parts of the descending pain inhibitory system involve dopaminergic pathways (i.e., in the rostral agranular insular cortex and dorsal horn neurons) [4, 9], dysregulation of dopaminergic transmission might contribute to altered pain processing in PD.

Conditioned pain modulation (CPM) paradigms, in which pain intensity ratings of noxious test stimuli are obtained in the presence and absence of a concomitantly presented conditioning stimulus to a remote body part [53], represent a well-established way to study descending pain inhibition in humans. Positive CPM responses (i.e., reduced pain intensity ratings under concurrent stimulation) are indicative of endogenous analgesia and are mediated by spino-bulbo-spinal reflexes [32], which are controlled by higher cortical brain areas [34, 41, 46].

To date, there are only few studies investigating CPM responses in patients with idiopathic Parkinson reporting no significant differences in CPM responses in medicated PD patients as compared to healthy controls [22, 23, 35]. Furthermore, over-night withdrawal of medication had no effect on CPM responses in PD patients [22, 23]. However, this washout phase might have been too short to sufficiently eliminate the dopaminergic medication. Although the plasma half-life period of dopamine agonists is relatively short (usually several hours) [7] and that of levodopa is generally estimated as 0.7 to 1.4 h [11], the latter can last up to 7.9 days [15]. The residual dopaminergic concentration might therefore still have induced antinociceptive effects [4] and could have normalized a reduced CPM response in PD patients. A recent study in patients with restless leg syndrome suggested that antinociceptive/analgesic effects of dopamine are concentration-dependent [4]. Low dopaminergic concentrations induced antinociceptive effects via dopaminergic D2 receptors, whereas higher levels had pro-nociceptive effects based on the activation of D1 receptors [4, 40]. Dopamine could therefore either increase or decrease CPM responses in PD patients depending on its concentration and low concentrations might have led to decreased pain during the insufficient washout phase.

In order to avoid this and other treatment-related confounds, we studied CPM responses using an established paradigm [23, 24, 46] in drug-naïve de novo PD patients. Specifically, we investigated whether CPM responses differed between de novo PD patients and healthy controls. On the assumption that distinct patterns of neurodegeneration underlying different PD subtypes [39] might affect pain modulation differentially, we also assessed the influence of the PD subtype on CPM responses, which has not been assessed in previous studies.

## Methods

### Participants

The study was conducted between 2011 and 2013 at the Department of Neurology at the University Medical Center Hamburg.

De novo PD patients were recruited from our movement disorders outpatient clinic (head: Prof. Carsten Buhmann) and had to fulfill the following inclusion criteria: (1) diagnosis of idiopathic PD according to the criteria of the UK PD Society Brain Bank, (2) Hoehn & Yahr scale ≤ stage III [27], (3) naïve to dopaminergic medication (“de novo” patients), (4) age between 40 and 90 years, (5) no severe cognitive impairment (Parkinson Neuropsychometric Dementia Assessment (PANDA; [28]) instrument ≥15), (6) no manifest depression or anxiety, (7) no acute pain or analgesic medication during the last 24 h, (8) no history of chronic pain disorders e.g. rheumatoid arthritis (PD specific chronic pain [16] was allowed), (9) no regular use of prescription analgesics, tranquilizers, antidepressants, pain modulating anticonvulsants (e.g. gabapentin or pregabaline), and (9) no pregnancy.

Healthy controls matched for age (+/− 3 years) and gender were recruited locally by announcement and had to fulfill the same inclusion criteria except for (1) and (2). This matched control group of the same size was used to calculate group comparisons to reveal potential abnormalities of measured parameters in the de novo group.

Out of the initially recruited sample size of 25 patients and 24 healthy controls, eight patients and seven healthy controls had to be excluded due to not fulfilling the inclusion criteria mentioned above or withdrawal from the study during the exposure to ice water. In detail, one patient decided to withdraw from the study, six had started dopaminergic medication at the day the study was performed, and one patient and seven healthy controls withdrew during the exposure to ice water. Only participants who finished the study were included into data analysis.

### Experimental protocol

In this study, we used an established CPM paradigm [23, 24, 46], which combines painful heat stimuli as test stimuli (TS) with a cold pressor task as the conditioning stimulus (CS). Patients were informed about the study, its purpose, and the study protocol using a standardized protocol after a regular visit in our outpatient clinic. After answering their questions, inclusion criteria were checked and written information material was handed out.

**Fig. 1:**
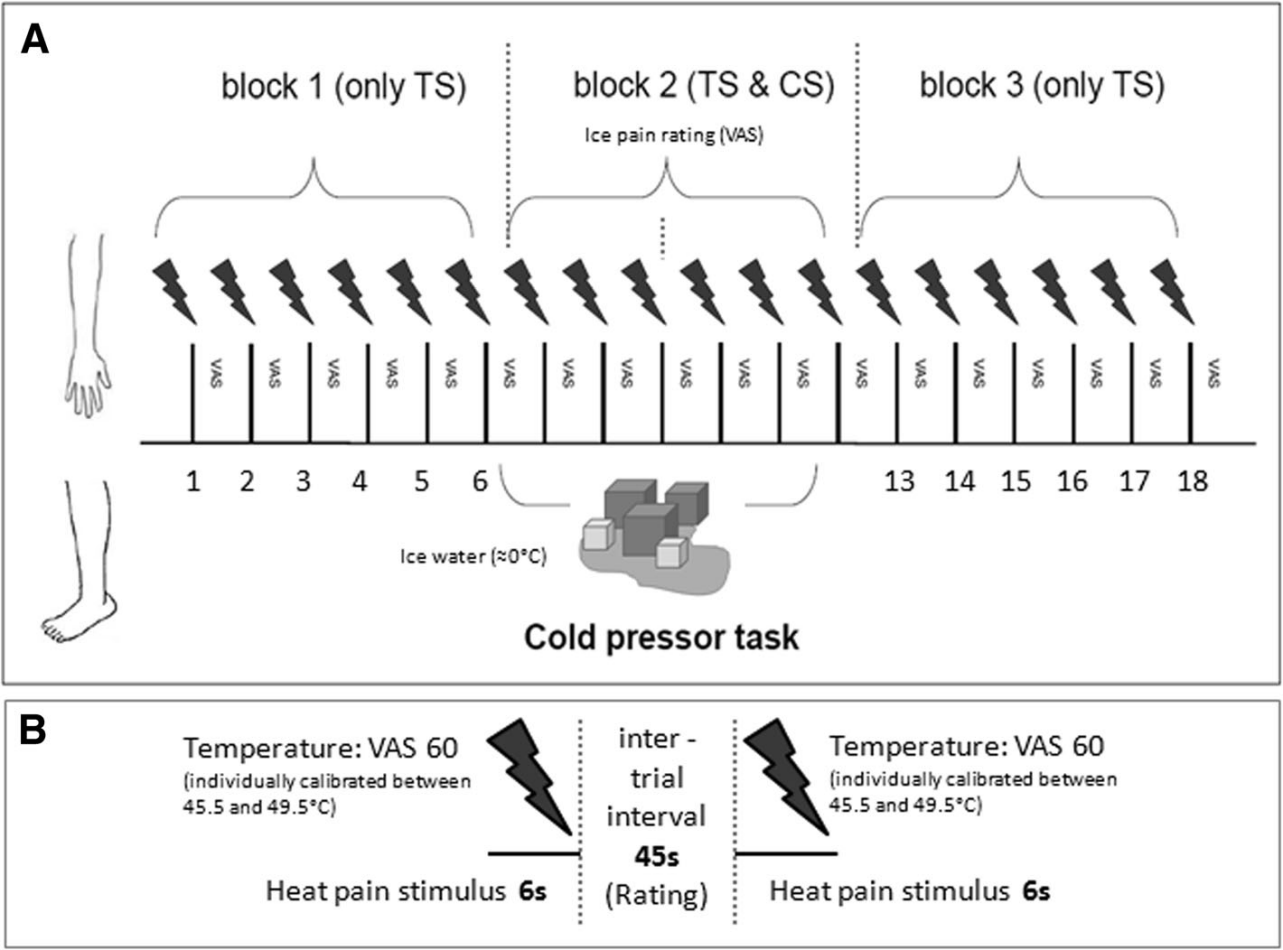
Experimental sequences of the CPM paradigm used in this study. (**a** whole experiment, **b** temporal components of trials). During block 1 and 3, the test stimulus (TS) was applied alone, whereas during block 2, the TS and a conditioning stimulus (= cold pressor task using ice water; CS) were applied concurrently. Subjects had to rate the pain intensity of TS and CS on a visual analogue scale (VAS)

In brief, the experimental procedures included an introductory session, which consisted of a clinical interview to check again all aforementioned inclusion criteria and re-evaluate the PD diagnosis, the assessment of Hoehn and Yahr stage, Unified Parkinson’s Disease Rating Scale and PD subtype, filling in of questionnaires (see Assessment of anxiety and depression), and the calibration of stimulus intensities (see Instructions and calibration procedure). This was followed by the a priori assessment of expectation ratings regarding possible changes of pain intensities during the application of the cold pressor task. Finally, the actual CPM paradigm was performed. The paradigm consisted of three blocks, in which six test stimuli each were applied to the right volar forearm. Pain ratings to these stimuli were obtained before (=block I), during (=block II) and after (=block III) a cold pressor task that was applied to the contralateral left leg during the second block. The experimental protocol is summarized in Fig. 1. All experimental parts were performed on the same day in one single session usually a few days (weeks) after patients had been informed about the study in our outpatient clinic.

#### Instructions and calibration procedure

All participants were instructed using a standardized protocol. Participants were told that the purpose of the study was to characterize possible differences in the perception of two simultaneously applied painful stimuli comparing PD patients with healthy participants of the same age. First, participants were informed about the sequence of experimental procedures. These general instructions were followed by a clinical interview (both groups) checking all inclusion criteria. In the PD group, an experienced clinician re-evaluated the PD diagnosis and assessed the individual Hoehn and Yahr stage [26], Unified Parkinson’s Disease Rating Scale (UPDRS [14]) score (total and motor score (part III)), and PD subtype. The PANDA [28] tested for cognitive impairment. Subjects with scores < 15 were excluded to ensure a sufficient task comprehension and compliance. PD subtypes were classified clinically according to the German AWMF Guidelines (www.awmf.org) as tremor-dominant (*n* = 9), akinetic-rigid (*n* = 5) or mixed (*n* = 3) depending on the predominant motor symptom (tremor, bradykinesia/rigidity or an equal manifestation of both) that had to be predominant at symptom onset and over the course of disease. Both groups completed the HADS [56] as depression and anxiety can modulate pain perception [1, 10] and acute pain was assessed asking the patients for any pain they might have experienced during the 24 h prior to the experiment (in case subjects experienced pain during 24 h prior to the experiment subjects were excluded from the study). If subjects fulfilled the inclusion criteria (see Participants) a calibration procedure was performed to determine the individual temperatures corresponding to a pain level of 50–60 on a 0–100 visual analogue scale [VAS, endpoints 0–100]. To this end, we applied 10 stimuli á 6 s each with different intensities ranging from 45.5–49.5 °C in a pseudo-randomized order to the right volar forearm, every temperature was presented once. Participants were asked to rate the intensity of each stimulus on a VAS, which was presented on a computer screen in front of the subjects and ranged from 0 = “no sensation” to 100 = “most intense pain imaginable”. Two vertical white lines represented the two endpoints 0 and 100 of the VAS, a third white line was set at 25 labeled as “pain threshold” to assess non-painful sensations, which might occur during the cold pressor task as a result of an effective pain inhibition. Subjects indicated the pain intensity of each heat pain stimulus by moving a red bar between the two endpoints using two buttons of a computer mouse. Participants did have as much time as they needed to provide their ratings, the experiment continued afterwards. The maximum stimulation temperature was restricted to 49.5 °C in order to avoid any tissue damage. The calibration procedure ensured that all participants perceived the phasic heat pain stimuli (= test stimuli, TS) as comparably painful (VAS 50–60).

The application of the thermal stimuli, the presentation of the VAS and the recording of behavioral data was performed using the software “Presentation®” (www.neurobs.com).

#### Test stimulus

We used phasic heat pain stimuli as test stimuli (TS). The test stimuli were applied to the right volar forearm (~ 10 cm proximally from the wrist) using a 30x30mm Peltier-Thermode (TSAII, Medoc, Israel). Each stimulus had a duration of 6 s (baseline temperature 35 °C, ramp up and down 10 °C/second, destination temperature individually calibrated between 45.5 and 49.5 °C, interstimulus-interval ~ 45 s). Pain ratings on the VAS were obtained immediately after each stimulus. A total of 18 test stimuli were applied. The first (=*block I*, stimulus 1 to 6) and the last six stimuli (= *block III*, stimulus 13 to 18) were applied without any other concomitant procedures. During the application of test stimuli 7 to 12 (=*block II*), the conditioning stimulus was applied.

#### Conditioning stimulus

A cold pressor task was used as the conditioning stimulus (CS). After completion of the first block of 6 heat pain stimuli (*block I*), a message on the computer screen prompted the participants to immerse their left foot into a bath with ice water (~ 0 °C). The intensity of the conditioning stimulus was rated once in the middle of the cold pressor task (= after TS 9, block II) using a VAS presented on a computer screen with the same endpoint labels 0 = “no sensation” and 100 = “most intense pain imaginable” and a third white line set at 25 labeled as “pain threshold”. At the end of block II, another message on the computer screen instructed the participants to take their foot out of the ice water. After taking their foot out of the ice water, participants positioned their foot in a towel on the floor next to the tub with ice water. Prior to the experiment, subjects were asked to focus their attention on the heat stimuli applied to the arm while having their foot immersed into the ice water and it was pointed out again that they could withdraw from the experiment at any time by telling the supervising experimenter. Finally, heat pain stimuli 13 to 18 (block III) were applied without concomitant painful stimulation to the foot.

#### Assessment of individual expectation

Many cognitive and affective processes could influence CPM responses. However, expectations which are known to modulate pain have previously been suggested to affect CPM responses [12, 21, 31, 36]. Following the calibration procedure, prior to the actual experiment, patients were presented the following question on the computer screen: “How do you expect the pain applied to your arm to change while you have your foot immersed into the ice water?” Participants were asked to indicate their expectations on a computerized VAS with the verbal anchors − 1 = “no sensation” (=pain at the arm would be completely abolished during the cold pressor task), 0 = “no change” (=no change of heat pain at the arm during the cold pressor task), and 1 = “maximum pain” (=pain applied to the arm would get worse during the cold pressor task). Two vertical white lines represented the two endpoints − 1 (“no sensation”) and 1 (“maximum pain”) of the VAS, a third white line was set at 0 labeled as “no change”. Subjects indicated their expectation by moving a red bar between the two endpoints using two buttons of a computer mouse. Participants did have as much time as they needed to provide their ratings. As in previous studies, no specific suggestions regarding the direction of possible changes were divulged [12].

#### Assessment of anxiety and depression

The Hospital Anxiety and Depression Scale (HADS) [56] is a self-report questionnaire to assess anxiety and depression with 7 items per subscale. Each item is scored from 0 to 3 points so that scores of 21 points for each subscale depression and anxiety can be reached. Higher scores indicate higher symptom severity. Both subscales have been validated to have good sensitivity and specificity [5].

### Data analysis

Data analysis was performed using IBM SPSS 20.0. There was no missing data. Non-parametric tests were used in case the assumptions of variance homogeneity (Levene’s test) and normal distribution (Kolmogorov-Smirnov test) were violated. For between-group comparisons between patients and controls, we used two-sample t-tests and non-parametric Mann-Whitney U tests.

CPM responses were calculated as the difference between mean pain ratings before and after the CS and mean pain ratings during the cold pressor task (CPM response = (mean pain ratings of blocks (1 + 3)) – (mean pain rating of block 2)) as described in previous studies [23, 24, 46]. A positive CPM response indicates a reduction in pain perception during the cold pressor task and therefore signifies analgesia symbolizing effective descending pain inhibition mechanisms, whereas a negative CPM response shows an increase of pain ratings in block II.

As different methods to calculate CPM responses are described (for review see [43, 54]), we also analyzed descending pain inhibition mechanisms in our participants using two other methods:

- block 1 – block 2: possible differences between patients and controls regarding mean pain ratings of test stimuli before (=block 1) and during CS (= block 2)
- block 1 mean (stimulus1,2,3) – block 2 mean (stimulus1,2,3): possible differences between patients and controls regarding mean pain ratings of the first 3 test stimuli (mean of block 1 stimulus 1,2,3) and the first 3 stimuli of block 2 (mean of block 2 stimulus 1,2,3).

This data is presented in the Additional file 1.

To test for group-specific CPM responses in healthy controls and patients, separate one sample t-tests on CPM responses were performed for the PD group and the control group. Kruskal-Wallis tests were used for PD subtype analyses. Correlations were calculated using Pearson’s (r-value) or non-parametric Spearman’s coefficients (rho-value). *P*-values < 0.05 were considered as statistically significant. Test results were corrected for multiple comparisons using Bonferroni correction.

## Results

### Clinical and neuropsychiatric assessment

Seventeen de novo PD patients (mean 61.9 years +/− standard deviation (SD) 9.8, range 42–75) and 17 healthy controls (61.7+/− 9.8, range 45–75) matched in age (*t(32) = − 0.070, p = 0.945*) and gender (each group: 10 male, 7 female) were included in the study. PD patient characteristics are given in Table 1. All group comparisons between patients and controls are given in Table 2. Mean PANDA scores differed significantly between both groups with lower PANDA scores in the patient group. Mann-Whitney U tests revealed a significant difference between the groups in the HADS depression subscore with higher scores in the patient group. HADS anxiety scores were comparable between both groups.

**Table 1:** Clinical characteristics of de novo Parkinson patients

Parameters	Patient characteristics (*n* = 17) mean +/− standard deviation [Min - Max]
Age	61.9 years +/− 9.8 [42–75 years]
Gender	10 male, 7 female
Handedness	16 right-handed, 1 left-handed
Hoehn & Yahr Scale (H & Y)	- H & Y stage I: 4 patients - H & Y stage II/ II.5: 12 patients - H & Y stage III: 1 patients
PD subtype	- akinetic-rigid: 5 patients - tremor-dominant: 9 patients - mixed type: 3 patients
Clinically most affected half of the body	- right: 4 patients - left: 13 patients
Disease duration (months since first time occurrence of symptoms prior to study)	18 months +/− SD 15.8 [4–60 months]
Unified Parkinson’s Disease Rating Scale (UPDRS) – total score	30.4 +/− 11.6 [15–48]
Unified Parkinson’s Disease Rating Scale (UPDRS) – motor score	21.2 +/− 8.3 [9–33]

**Table 2:** Main results for healthy subjects and de novo Parkinson patients (PD) in group comparison

Parameters (mean, standard deviation, [Min-Max])	Healthy subjects (*n* = 17)	De novo Parkinson patients (*n* = 17)	Group comparison Healthy subjects vs. De novo (*p*-value)
Age	61.7+/− 9.8 [45–75]	61.9 +/− 9.8 [42–75]	t(32) = − 0.070, *p* = 0.945
Gender	10 male, 7 female	10 male, 7 female	**–**
PANDA Score	24.6 +/− SD 3.0 [17–29]	21.1 +/− 4.0 [15–29]	**t(32) = 2.848,** ***p*** **= 0.008**
HADS subscale depression	1.3 +/− 1.3 [0–4]	3.5 +/− 2.5 [0–9]	**U = 227.5, z = 2.901,** ***p*** **= 0.003**
HADS subscale anxiety	2.2 +/− 2.1 [0–6]	4.5 +/− 4.3 [0–14]	U = 187.5, z = 1.498, *p* = 0.140
Mean stimulation temperature	48.2 +/− 0.8 [47.0–49.5]	47.9 +/− 0.9 [45.5–49.0]	U = 80.5, z = − 0.739, *p* = 0.478
Mean pain intensity Block 1 [VAS 0–100]	55.9 +/− 8.8 [38.7–67.0]	60.8 +/− 12.1 [37.8–86.8]	t(32) = − 1.380, *p* = 0.177
Cold pain rating Block 2 [VAS 0–100]	74.8 +/− 18.3 [29–97]	58.7+/− 27.4 [14–96]	t(27.874) = 2.023, *p* = 0.053*
Expectation Rating Day 1 [− 1 to + 1]	− 0.2 +/− 0.4 [− 0.8–0.5]	0.1 +/− 0.4 [− 0.7–0.9]	**t(32) = −2.609,** ***p*** **= 0.014**
CPM Magnitude	3.1 +/− 6.4 [− 5.1–13.3]	1.7 +/− 6.7 [− 9.8–13.9]	t(32) = 0.611, *p* = 0.545

Significant results are marked in bold

*Abbreviations*: *UPDRS* Unified Parkinson’s disease rating scale, *HADS* Hospital anxiety and depression score, *PANDA* Parkinson neuropsychometric dementia assessment

*= *P*-value and degree of freedom corrected for unequal variances

### Experimental parameters and expectation ratings

Stimulation temperatures and mean pain ratings in block 1 did not differ significantly between groups (see Table 2). Mean cold pain ratings only differed by trend (*p = 0.053*) with slightly higher pain ratings in controls. Interestingly, expectation ratings differed between both groups. De novo patients expected the heat pain to be similar or slightly increased under concurrent ice stimulation, whereas controls expected it to be reduced. There was no significant correlation of expectation with any of the clinical scores (PANDA, HADS_A, HADS_D, UPDRS total and motor score and symptom duration) or with CPM responses when pooling across groups and when analyzing these correlations for each group separately.

### CPM responses

**Fig. 2:**
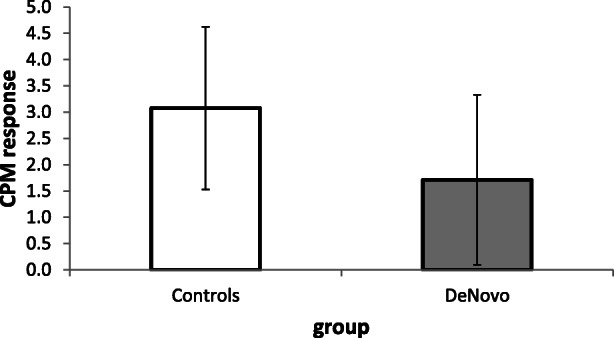
Conditioned pain modulation magnitudes are shown for healthy controls and de novo PD patients. Means and standard error of means are presented. There were no group differences between de novo patients (gray bar) and controls (white bar)

CPM responses did not differ between controls and patients (*p = 0.545*, see Table 2 and Fig. 2). Analyses of group-specific CPM responses using one sample t-tests revealed that neither patients (1.7+/− 6.7; *t(16) = 1.058, p = 0.306*) nor controls (3.1+/− 6.4; *t(16) = 1.989, p = 0.064*) showed a significant CPM response.

CPM responses of the alternative calculation methods (a) block 1 – block 2 and b) block 1 mean (stimulus1,2,3) – block 2 mean (stimulus1,2,3)) are presented in the Additional file 1.

#### CPM responses and PD subtypes

**Fig. 3:**
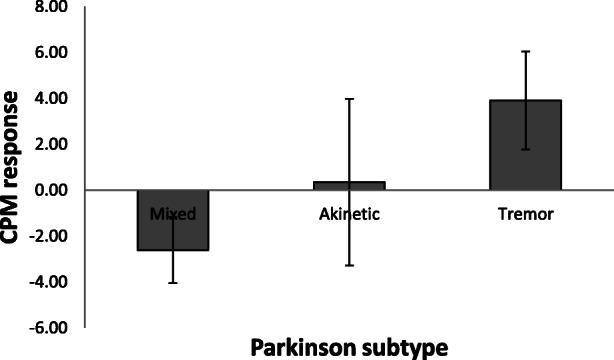
Conditioned pain modulation magnitudes are shown for Parkinson subtypes. Means and standard error of means are presented. There were no significant subtype differences

Kruskal-Wallis tests revealed no significant differences between the 3 subtypes with respect to age, UPDRS scores, PANDA scores, the HADS subscales for anxiety and depression, stimulation temperature, mean pain intensity rating in block 1 and cold pain ratings. Tremor-dominant patients showed greater CPM responses (*n* = 9: 3.9+/− 6.4 [− 5.1–13.9]) compared to akinetic-rigid (*n* = 5: 0.4+/− 8.1 [− 9.8–10.8]) and mixed subtype PD patients (*n* = 3: − 2.6+/− 2.5 [− 4.3–0.3]; see Fig. 3). However, differences between groups did not reach statistical significance *(H(2) = 2.287, p = 0.319).* One sample t-tests revealed no significant CPM response in none of the subtypes (all *p > 0.100*).

#### Correlations of CPM responses with other parameters

There were no correlations between CPM responses and other parameters investigated in this study neither in patients nor in controls. As expected, in the patient group, UPDRS total and motor score correlation (*r = 0.968, p < 0.001*) as well as HADS_A and HADS_D correlation were very high (*rho = 0.801, p = 0.000*).

## Discussion

In this study, we used a well-established CPM paradigm to investigate endogenous pain modulation in drug naïve de novo PD patients. Our results do not show any significant differences between CPM responses of de novo PD patients and healthy subjects matched for age and gender, indicating no additional impairment of descending pain inhibition in PD besides the known age-related decline in CPM responses [13, 24, 31, 52]. In this study, we have chosen an established CPM paradigm that has been applied in previous studies [23, 24, 46]. However, several different methods to calculate CPM response are described [43, 54]. Here, using two alternative calculation methods revealed the same result, i.e. similar CPM responses in de novo PD and healthy controls (see Additional file 1). However, it should be noted that we did not find a significant CPM effect in the healthy controls.

Our finding is in line with previous CPM studies in medicated PD patients [22, 23, 35] reporting no differences in CPM responses between PD patients undergoing temporary withdrawal from dopaminergic medication and healthy controls. This result should be interpreted cautiously with view to the relatively small sample sizes in our and in previous studies. Nevertheless, together, these findings suggest that neither dopaminergic degeneration per se nor the dopaminergic anti-parkinsonian medication have relevant effects on descending pain inhibition in PD patients, which renders it unlikely that the high prevalence of pain in PD [3] is the result of impaired descending pain modulation. However, the CPM paradigm used in our study is only one experimental way to assess the capacity for endogenous pain control and non-significant findings cannot rule out neurobiological differences in this system between PD patients and healthy controls.

It is also possible that the lack of CPM response differences between PD patients and healthy controls results from pooling PD patients across subtypes, which might vary with respect to endogenous pain control. In order to investigate whether the capacity for endogenous pain modulation varied between PD subtypes, we compared CPM responses between patients with tremor-dominant, akinetic-rigid and mixed type PD. Although differences between groups failed to reach statistical significance, it seems noteworthy that CPM responses were stronger in the tremor-dominant type than the akinetic-rigid and mixed type.

As musculoskeletal pain, the most common painful sensation in PD [17] with prevalence up to 70% [3], is frequently associated with rigidity [17], akinetic-rigid patients might hypothetically be more likely to suffer from chronic pain than other subtypes. Given that these patients are also more impaired due to faster disease progression, higher frequency of motor fluctuations, a greater risk of cognitive dysfunction [2] and depression [47] compared to other PD subtypes, it is conceivable that the more extensive neurodegeneration in the akinetic-rigid subtype [39] also involves brain areas relevant for pain processing and modulation. Future studies on larger patient samples should therefore further explore potential subtype differences in CPM responses. As it is conceivable that CPM subtype differences might develop over the time and with disease progression, these future studies should also include PD patients with longer disease durations where potential subtype differences might be more pronounced. Moreover, in future studies with larger sample sizes, a Bayesian statistical approach would represent an appropriate way to provide more insights into potential group differences, or their absence, and PD subtypes. For that, bigger sample sizes are needed. We are aware that our subgroups are very small and that our results are not statistically significant, yet by publishing this data, we hope to draw attention to this topic of possible differences between PD subtypes.

Given the absence of significant CPM response differences between PD patients and healthy controls, abnormalities in other mechanisms involved in pain perception or pain processing in PD might be more conclusive to explain clinical pain in PD. For instance, there is first evidence for an abnormal central processing of nociceptive input [48] and abnormal brain activation in areas involved in pain processing in PD [6]. The attenuation of pain during deep brain stimulation of central brain structures, such as the globus pallidus or nucleus subthalamicus [30], also supports the hypothesis of altered central pain processing in PD. However, changes in structures such as epidermal nerve fibres / Meissner corpuscles [37] or unmyelinated nerve fibers [29] also point towards a contribution of the peripheral nervous system.

The temporal profile of pain in PD is highly variable with patients reporting pain even prior to the occurrence of motor symptoms [45, 50] on the one hand and pain - especially dystonic pain [20] - developing under dopaminergic medication [17, 45] on the other hand. This indicates that pain due to dopaminergic degeneration and pain resulting from anti-parkinsonian medication might be based on different mechanisms. Furthermore, dopaminergic medication shows different effects on different types of pain in PD. For instance, levodopa increases dystonic pain [49], but improves musculoskeletal pain [25]. Future studies should therefore differentiate between different types of pain in PD (e.g. according to Ford et al. [17]) in order to investigate etiological mechanisms underlying the different types of pain.

Finally, also handedness or disease-specific body side dominancy might have influenced our results. As we used a standardized protocol, test stimuli were by defaults applied to the right arm and conditioning stimuli to the contralateral left leg. However, other researchers have used the right body side for stimulation [19] without explicitly looking at handedness or disease-specific body side dominancy. Therefore, future studies should incorporate a both-sided testing to also assess a) the influence of handedness / hand-dominancy, since nearly all of our patients were right-handed, which has been described to potentially affect experimental pain sensitivity [43] and b) the impact of the most affected body side (13 out of 17 patients were most effected on their left side) as one previous study has found an increased sensory response causing hyperalgesia in patients with predominantly left-sided PD after dopaminergic medication [22].

It should further be noted as a limitation to our study that unfortunately, after we had already obtained the data for this study, new recommendations regarding protocols for testing CPM have been published, which should be applied in future studies [55]. However, our study still strictly followed a previously published standardized protocol.

## Conclusion

Our study, which is the first to study descending pain inhibitory mechanisms in de novo PD patients, provides further evidence against the assumption that PD is associated with a general deficit in pain regulation beyond the known age-related decline and suggests that mechanisms other than abnormalities in descending pain inhibition might explain the high pain prevalence in PD. Although differences between PD subgroups failed to reach statistical significance, stronger CPM responses in the tremor-dominant subtype point towards potential subgroup differences. Together, our results highlight the need for future studies in larger patient samples to elucidate the pathophysiological underpinnings of pain in PD.

## Additional file

Additional file 1: Supplementary Material. (DOCX 20 kb)

## Data Availability

The datasets used and analyzed during the current study are available from the corresponding author on reasonable request.

## Abbreviations

CPM: Conditioned pain modulation
CS: Conditioning stimulus
HADS: Hospital Anxiety and Depression Score
PANDA: Parkinson Neuropsychometric Dementia Assessment
PD: Parkinson’s disease
TS: Test stimulus
UPDRS: Unified Parkinson’s Disease Rating Scale
VAS: Visual analogue scale
