# Supplementary material for: Conditioned pain modulation in drug-naïve patients with de novo Parkinson’s disease
Source: Neurol Res Pract. 2019 Aug 26;1:27. doi: 10.1186/s42466-019-0029-x (PMC7650066; doi:10.1186/s42466-019-0029-x)
Supplement: Supplementary file 1 — Supplementary Material. (DOCX 20 kb) [file 42466_2019_29_MOESM1_ESM.docx]

**Supplementary Material**

**Conditioned pain modulation in drug-naïve patients with de novo Parkinson’s Disease**

Wiebke Grashorn^1^*, Odette Fründt^1^*, Carsten Buhmann^1^, Nathalie Wrobel^3^, Katharina Schmidt^2^, Ulrike Bingel^2^

* Both authors contributed equally to the manuscript.

^1^ Department of Neurology, University Medical Center Hamburg - Eppendorf, Martinistr. 52, 20246 Hamburg, Germany

^2^ Department of Neurology, University Hospital Essen, University Duisburg-Essen, Hufelandstrasse 55, 45147 Essen, Germany

^3^ Karolinska Institutet, 171 77 Stockholm, Sweden

**1.) Alternative Calculation Method 1 (block 1 - block 2)**

We additionally analyzed an alternative CPM calculation (block 1 - block 2) and investigated possible differences between block 1 vs. block 2 in both our patient and control group: We could not detect any significant differences between the patient and the control group (t(32)=-0.202, p=0.841).

In addition, analyses of group-specific alternative CPM responses (block 1 - block 2) using one sample t-tests revealed no significant CPM response in neither the patients (mean CPM response (block 1-block 2) = 2.3 +/-9.1, SEM 2.2; t(16)=1.056, p=0.307) nor the healthy controls (mean CPM response (block 1 – block 2) = 1.7 +/-9.9, SEM 2.4; t(16)=0.695, p=0.497). Mean pain ratings did also not differ between groups in block 1 (p=0.177) and block 2 (p=0.363).

**Figure S1**

*Sup. Fig. 1: Mean CPM responses for both groups using the alternative calculation method 1 (block 1 - block 2) are shown (left = control group, right = De Novo Parkinson patient group) and did not significantly differ between groups.*

**2.) Alternative Calculation Method 2 (mean of block 1 (stimulus 1,2,3)) – (mean of block 2 (stimulus 1,2,3)):**

We further calculated CPM responses using the first three ratings for the test stimulus and during application of the conditioning stimulus (= CPM 1.1-3 vs. 2.1-3 = ((mean of block 1 stimulus 1,2,3) – (mean of block 2 stimulus 1,2,3)). There were no significant differences of CPM responses using this calculation method between patient and control group: t(32)=-0.013, p=0.989.

**Figure S2**

*Sup. Fig. 2: Mean CPM responses for both groups using the alternative calculation method 2 (mean of block 1 (stimulus 1,2,3)) – (mean of block 2 (stimulus 1,2,3)) are shown (left = control group, right = De Novo Parkinson patient group) and did not significantly differ between groups.*

We also compared the mean pain ratings of the first three stimuli separately for block 1 and 2 between the two groups: There were no significant differences between groups neither for block 1 nor block 2 (mean block 1 stimuli 1-3: p= 0.076; mean block 2 stimuli 1-3: p= 0.197).
